# Physicochemical characteristics and metabolomic profiling of representative rice cultivars distinct in eating quality

**DOI:** 10.3389/fnut.2026.1914365

**Published:** 2026-08-17

**Authors:** Dongping Yao, Siyu Peng, Xiaomei Li, Xinjie Huang, Chaoming Xing, Jiangwei Yin, Bin Bai

**Affiliations:** 1 College of Plant Science and Technology, Hunan Biological and Electromechanical Polytechnic, Changsha, Hunan, China; 2 College of Agronomy, Hunan Agricultural University, Changsha, Hunan, China; 3 State Key Laboratory of Hybrid Rice, Hunan Hybrid Rice Research Center, Changsha, Hunan, China

**Keywords:** 2-acetyl-1-pyrroline, amylopectin long-to-short chain ratio, eating quality, metabolome, physicochemical properties, rice

## Abstract

This study aimed to identify shared physicochemical disparities between high eating quality rice and conventional high-yield cultivars across indica and japonica subspecies, and screen core taste-related trait indicators, while characterizing intrasubtype metabolic variation in indica rice. Four representative rice cultivars (two premium fragrant rice: Yuzhenxiang, Daohuaxiang No. 2; two high-yield rice: Huanghuazhan, Kendao 2066) were comprehensively evaluated via sensory, texture, RVA and multi-component quantitative detection; Untargeted metabolomics was further performed on the two indica cultivars Yuzhenxiang and Huanghuazhan. Rice with superior eating quality featured low protein, amino acids and citric acid, moderate soluble sugars, abundant 2-AP, elevated amylopectin long/short chain ratio and low gelatinization temperature. A total of 1,318 differential metabolites were identified, with arginine biosynthesis and linoleic acid metabolism as the most significantly enriched pathways. A three-indicator multiple linear regression model with *R*^2^ = 0.972 was constructed for preliminary quantitative prediction only within tested materials. Findings from this multi-indicator integrated analysis revealed that starch structure, aroma compound 2-AP and small-molecule flavor substances jointly regulate rice taste across subspecies; among them, 2-AP, amylopectin long/short chain ratio and RVA consistency viscosity act as core regulators of rice eating quality for both indica and japonica rice, while differential metabolites mainly account for taste variation within indica varieties. This study provides multi-dimensional theoretical references and candidate markers for high-quality rice breeding and quality evaluation.

## 1 Introduction

Rice (*Oryza sativa L*.) is one of the most important staple food crops in the world, providing the primary energy source for more than half of the global population. With socio-economic development and the upgrading of dietary structures, consumer demand for rice quality has shifted from the traditional goal of “high and stable yield” to the dual objective of “excellent eating quality and nutritional value”. Eating quality is a core indicator of the market acceptance and commercial value of rice, encompassing appearance, aroma, taste, and texture. Its formation is co-regulated by genetic background, environmental factors, and cultivation practices, and is jointly governed by grain physicochemical traits and endogenous metabolic substances.

In recent years, multi-dimensional analytical technologies including metabolomics have greatly facilitated the in-depth exploration of rice eating quality (1). Zhang et al. (2) showed that the eating quality of rice was closely related to the composition and dynamic changes of various metabolites in the grains. Shu et al. (3) applied UPLC-MS/MS-based metabolomics to 35 rice cultivars, identified 3,864 metabolites, and screened 10 potential markers significantly associated with eating quality; metabolites enriched in the glycerolipid metabolism pathway were positively correlated with taste, while those in the thiamine metabolism pathway showed a negative correlation. Kasote et al. (4) reported that volatile aroma components, such as 2-acetyl-1-pyrroline (2-AP), aldehydes, and alcohols, jointly shaped the aroma profile of cooked rice. Qi et al. (5) combined HS-SPME-GC-MS, GC-IMS, and sensory evaluation to classify rice aroma into three types (sweet popcorn, grainy/starchy, and complex), identified 74 volatile compounds, and pointed out that (E)-2-octenal, 6-undecanone, and 2-AP contributed to the sweet aroma characteristics of rice. Taste-related metabolites, such as free amino acids, soluble sugars, and organic acids, directly affected the basic taste profile of cooked rice (sweetness, umami, bitterness) (6).

More crucially, starch structural traits and grain storage proteins serve as the dominant physicochemical foundation shaping rice eating quality. Starch physicochemical parameters (especially amylose content and amylopectin chain-length distribution) and grain storage proteins and their composition represent another critical dimension of eating quality determinants. They influence pasting properties, texture, and retrogradation behavior, and together determine key textural attributes such as chewiness, stickiness, and elasticity, forming an important physicochemical foundation for eating quality (7).

Existing researches on rice eating quality generally have two obvious shortcomings. First, most studies only investigate a single type of index—either isolated metabolic compounds or separate starch indicators—lacking integrated multi-dimensional comparison combining sensory, instrumental texture and comprehensive physicochemical data. Second, most comparative analyses only focus on single rice subspecies, failing to systematically reveal shared and differential quality traits across indica and japonica. Accordingly, four representative rice cultivars were selected with a balanced design: one high-quality and one high-yield type for both indica and japonica subspecies, and multi-dimensional detection systems were constructed. All four cultivars were subjected to comprehensive sensory and physicochemical measurements to realize inter-subspecies comparison of eating quality between indica and japonica; untargeted metabolomics was only carried out on the two indica cultivars to reveal the metabolic differences between high-quality fragrant indica rice and conventional high-yield indica rice within the same subspecies. Volatile and non-volatile metabolic profiles were comprehensively characterized via sensory evaluation, taste analyzer detection, gas chromatography-mass spectrometry (HS-SPME/GC-MS) and ultra-high-performance liquid chromatography-high-resolution mass spectrometry (UHPLC-Q-Exactive MS). Through multivariate statistics and correlation network analysis, this work screens core indicators associated with superior eating quality, clarifies physicochemical formation rules of rice eating quality, and provides theoretical references and screening indices for the precise evaluation and molecular breeding of high eating quality rice in China.

## 2 Materials and methods

### 2.1 Plant materials and sample preparation

Four rice cultivars with significantly distinct eating quality were selected as experimental materials, covering two indica and two japonica subtypes: two premium fragrant rice cultivars with superior eating quality (Yuzhenxiang, YZX; Daohuaxiang No.2, DHX2), and two high-yield cultivars with moderate eating quality (Huanghuazhan, HHZ; Kendao 2066, KD2066). The two indica cultivars YZX and HHZ were planted and harvested in Hunan Province in the same year; the japonica cultivars DHX2 and KD2066 were obtained from field cultivation in Daqing, Heilongjiang Province. All materials were newly harvested mature paddy rice of the current year. After collection, all paddy grains were uniformly milled under consistent processing parameters to eliminate interference from inconsistent milling conditions.

Three biological replicates were prepared for each cultivar in routine physicochemical, texture and sensory analyses. For untargeted metabolomic profiling, five independent biological replicates were collected for YZX and HHZ only, while DHX2 and KD2066 were not subjected to metabolomics detection. After milling, the polished rice was refrigerated at 4 °C, and subsequent determinations were conducted until their physicochemical properties stabilized. Intact milled rice grains were directly cooked for human sensory evaluation and instrumental taste analyzer measurements. For physicochemical and metabolomics analyses, milled rice grains were ground using a cyclone sample mill (FOSS CT298) and sieved through a 100-mesh sieve to prepare rice flour for subsequent testing.

### 2.2 Instrumental taste value measurement

The taste value was determined using a Cooked Rice Taste Analyzer (STA1B, SATAKE, Japan) following the method described by Yao et al. (8). Briefly, 30 g of polished rice was weighed, placed in a stainless steel container, and soaked for 30 min. The rice was then rinsed with cold water until the rinse water became clear, and distilled water was added according to the ratio of rice to water 1:1.2 (w/w). The rice was cooked for 30 min and kept warm for 10 min. After cooling, 7 g of the cooked rice was pressed into a defined volume, and the comprehensive taste value was measured. Each sample was analyzed in triplicate.

### 2.3 Texture profile analysis (TPA) of cooked rice

The texture characteristics of cooked rice were objectively evaluated using a texture analyzer (TA.XT Plus, Stable Micro Systems, UK) in the Texture Profile Analysis (TPA) mode. The cooked rice samples used for TPA were prepared in the same batch as those for instrumental taste evaluation. After cooling to room temperature, 20.0 g of cooked rice was pressed using a rice presser with constant force for 30 s to form a cylindrical “rice cake” of uniform height and density. The texture analyzer was calibrated with standard weights before measurement. The rice cake was placed in the center of the instrument base, and a cylindrical flat-bottomed probe (P/36R) was used. The TPA test parameters were set as follows: two-compression TPA mode was selected; pre-test speed, test speed and post-test speed were all set at 50 mm/min; the compression deformation was defined as 50% of the original sample height; the interval between two compressions was set to 0 s, and the trigger force was 0.25 N. The TPA curves were automatically recorded by the accompanying software (Exponent), and parameters including hardness, adhesiveness, springiness, cohesiveness, and chewiness were calculated.

### 2.4 Sensory evaluation

Sensory evaluation was performed following the standardized panel operation described by Yao et al. (8), strictly complying with the requirements of Chinese national standard GB/T 15,682–2025. Briefly, rice samples were rinsed and soaked for 30 min; water was added at a baseline rice-to-water mass ratio of 1:1.4, with minor adjustments allowed according to rice texture performance, followed by steaming and simmering before blind tasting. Five to ten professionally trained rice sensory assessors finished the evaluation, covering five indicators: aroma, appearance, palatability, taste and cold rice texture. Outliers deviating over 5 points from the average were discarded, and the integer average score was taken as the final eating quality result of each cooked rice sample.

### 2.5 Determination of major physicochemical components

Total starch content was determined by acid hydrolysis according to the Chinese national standard GB 5,009.9–2023 “National food safety standard—Determination of starch in foods,” and rice starch quantification reported by Chen et al. (9) was cited as a general methodological reference. Apparent amylose content was determined using the iodine-binding spectrophotometric protocol from Zhang et al. (10). The distribution of amylopectin chain lengths was analyzed using an ICS5000 ion chromatography system (ICS-5,000, Thermo Fisher Scientific, USA) following the method described by Yao et al. (8). A Dionex CarboPac PA200 ion-exchange column (3.0 × 250 mm, Thermo Fisher Scientific, USA) was used. Amylopectin chains were classified into four types: A chain (6 ≤ DP ≤ 12), B1 chain (13 ≤ DP ≤ 24), B2 chain (25 ≤ DP ≤ 36), and B3 chain (DP ≥ 37). Crude protein content was determined by the Kjeldahl method according to the Chinese national standard GB 5,009.5–2025 “National food safety standard—Determination of protein in foods” (9) using an automatic Kjeldahl apparatus (Kjeltec 8400, FOSS, Denmark).

### 2.6 Analysis of non-volatile substances

Free amino acids were determined using an amino acid autoanalyzer (LA8080, Hitachi, Japan) following the instrumental protocol described by Wang et al. (11). Fructose, glucose, sucrose, maltose, and lactose were determined were extracted by ultrasonic treatment and quantified via high-performance liquid chromatography (HPLC) using a Shimadzu LC-20AT system according to the method described by Yao et al. (12). According to Kang et al. (13), organic acids were determined using LC-MS/MS MRM analysis. Rice powder was extracted under heating reflux in 50% methanol with deuterated succinic acid as internal standard. After centrifugation and 0.22 μm filtration, analytes were separated on an HSST3 column via gradient elution of 0.1% formic acid-water and methanol. Electrospray negative ion mode was used for MS detection, and internal standard curves were established for quantification. GB/T 40,179–2021 was consulted to optimize chromatographic and MS conditions. Fatty acid composition of milled rice was determined via alkaline saponification and boron trifluoride methylation according to Ye et al. (14), referring to the first method of GB 5,009.168.

### 2.7 Untargeted metabolomics analysis

Untargeted metabolomics analysis was performed on two cultivars (HHZ and YZX) to explore intrasubtype metabolic divergence between high eating quality and conventional high-yield indica rice, using liquid chromatography-mass spectrometry (LC-MS) on a Q Exactive™ HF platform, and the experiment was conducted by Beijing Novogene Bioinformatics Technology Co., Ltd (Beijing, China). Each cultivar contained five independent biological replicates, and mixed pooled QC samples were arranged in the injection sequence to monitor instrument stability. Chromatographic separation adopted a BEH C18 column (2.1 × 100 mm, 1.7 μm) maintained at 40 °C with a flow rate of 0.2 ml/min; mobile phase A was 0.1% aqueous formic acid and mobile phase B was methanol. The Q Exactive™ HF mass spectrometer ran under dual positive/negative ESI ionization modes, with full scan m/z range of 70–1,000, MS resolution of 70,000 and MS/MS resolution of 17,500. The workflow included metabolite extraction, LC-MS/MS analysis, and data processing. Differential metabolites were screened based on the criteria: VIP > 1.0, fold change (FC) > 1.5 or FC < 0.667, and *P*-value < 0.05. Metabolites were annotated against HMDB, KEGG and LIPIDMAPS databases, while the METLIN library was used for supplementary MS/MS spectral matching to enhance identification reliability.

### 2.8 Determination of starch pasting viscosity properties

The pasting viscosity characteristics of starch were determined using a Rapid Visco Analyzer (RVA) to evaluate the dynamic changes in viscosity during heating and cooling. The measurement was performed following the measurement procedure reported by Yao et al. (15). Samples were determined in triplicate. The RVA profile characteristic parameters include: Peak Viscosity (PV), Trough Viscosity (TV), Breakdown Viscosity (BD), Final Viscosity (FV), Setback Viscosity (SB), Consistency Viscosity (CS), and Gelatinization Temperature (GT). The BD was calculated by subtracting the TV from PV. The SB was obtained by subtracting the PV from the FV, while the CS was acquired by subtracting the TV from the FV.

### 2.9 Statistical analysis

All chemical and instrumental analyses were performed with three biological replicates, and each replicate was measured three times. Results are presented as mean ± standard deviation. One-way analysis of variance (ANOVA) followed by Tukey's test (*p* < 0.05) was performed using SPSS 24.0 software. Pearson correlation analysis was used to analyze the relationship between taste value and major physiological indicators. Multivariate statistical analyses, including principal component analysis (PCA) and partial least squares discriminant analysis (PLS-DA), were applied to screen differential metabolites between superior and ordinary eating quality rice. The differential metabolite data were further integrated with taste value, texture profile, and protein content data through correlation network analysis to identify key metabolites most closely associated with the superior eating quality phenotype. Multiple linear regression was adopted to quantify the internal correlation of core indicators within tested materials.

## 3 Results and discussion

### 3.1 Comprehensive evaluation on eating quality of different rice cultivars

#### 3.1.1 Sensory evaluation scores of the four rice cultivars

Sensory evaluation of cooked rice was carried out following the standardized panel operation procedure described by Yao et al. (8), with reference to the unified scoring framework specified in the Chinese national standard GB/T 15,682–2025. Eight trained panelists evaluated four rice cultivars in terms of five sensory indicators: odor, appearance, palatability, taste, and cold rice texture. The results were presented in Table 1. The comprehensive sensory scores of the four tested rice cultivars were ranked as follows: YZX > DHX2 > HHZ > KD2066.

**Table 1:** Sensory evaluation scores of the four rice cultivars.

Rice sample name	Panel evaluation scores					
	Odor (20 points)	Appearance & structure (20 points)	Palatability (30 points)	Taste (25 points)	Cold rice texture (5 points)	Comprehensive sensory score (100 points)
DHX2	15.7^b^	17.3^a^	25.5^b^	22.3^a^	3.9^b^	85^b^
KD2066	14.6^c^	13.3^c^	20.4^c^	19.8^c^	2.9^c^	71^d^
YZX	17.6^a^	17.0^a^	26.4^a^	21.7^ab^	4.3^a^	87^a^
HHZ	13.4^d^	14.5^b^	21.1^c^	20.9^bc^	3.1^c^	73^c^

Values are means of scores from eight trained panelists. Different lowercase letters within the same column indicate significant differences among rice cultivars at *p* < 0.05, according to Tukey's multiple range test.

Significant differences were detected for all sensory indices among the four tested rice cultivars. YZX obtained the maximum comprehensive sensory score, ranking first owing to its top-ranked odor, excellent palatability, and optimal cold rice texture, demonstrating superior eating quality. DHX2 obtained the second-highest total score of 85, with acceptable odor, appearance and structure, palatability, and taste, possessing favorable sensory characteristics as premium rice. By comparison, HHZ and KD2066 exhibited markedly lower comprehensive sensory scores. Both cultivars scored poorly in odor, appearance and structure, and palatability; KD2066 presented the lowest values in appearance and structure, as well as cold rice texture, with HHZ having the minimum odor score, resulting in poor comprehensive sensory performance.

#### 3.1.2 Determination of taste value using a rice taste analyzer

**Figure 1:**
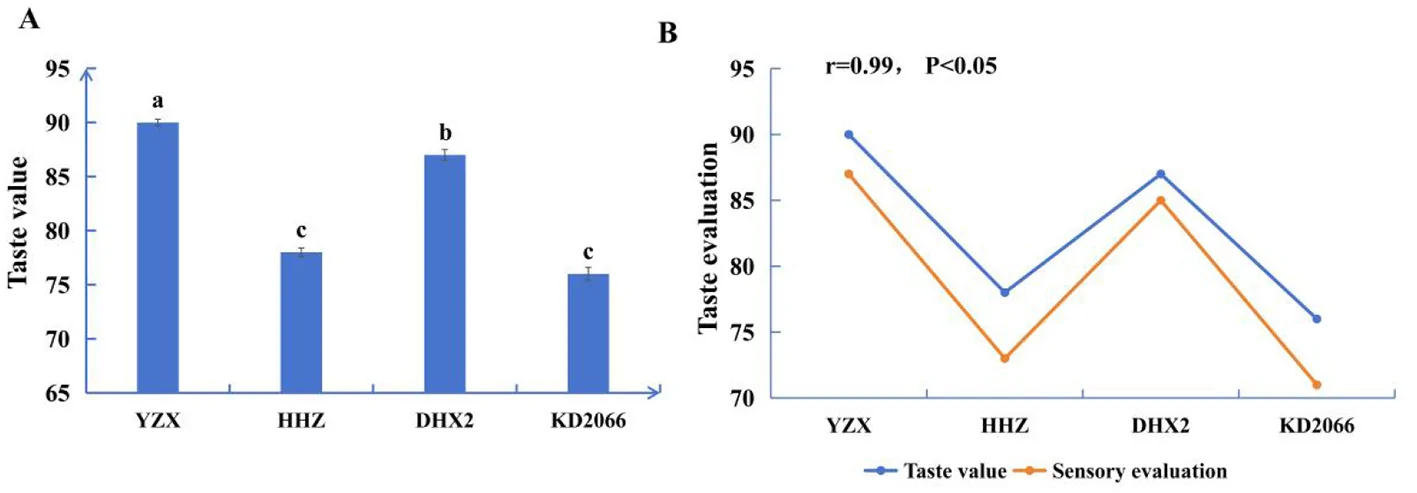
Taste values of four rice samples measured using the rice taste analyzer **(A)**, and the correlation between instrumental taste values and sensory evaluation scores **(B)**. Means with different lowercase letters in **(A)** indicate significant differences at *p* < 0.05. YZX (Yuzhenxiang), HHZ (Huanghuazhan), DHX2 (Daohuaxiang No.2), KD2066 (Kendao 2066).

A rice taste analyzer was used to determine the taste values of the four milled rice samples. As shown in Figure 1A, the highest taste value was recorded for YZX (90), followed by DHX2 (87); in comparison, HHZ (78) and KD2066 (76) exhibited significantly lower values. DHX2 scored significantly higher than HHZ and KD2066 (*p* < 0.05). No significant difference was found between HHZ and KD2066.

The correlation between instrumental taste values and the sensory evaluation scores provided by the panelists for the four rice cultivars was further analyzed (Figure 1B). Pearson correlation analysis revealed a strong and significant positive correlation between the two indicators (*r* = 0.99, *p* < 0.05), demonstrating that the taste value measured by the instrument could effectively reflect the sensory evaluation results provided by trained panelists.

#### 3.1.3 Textural properties

**Figure 2:**
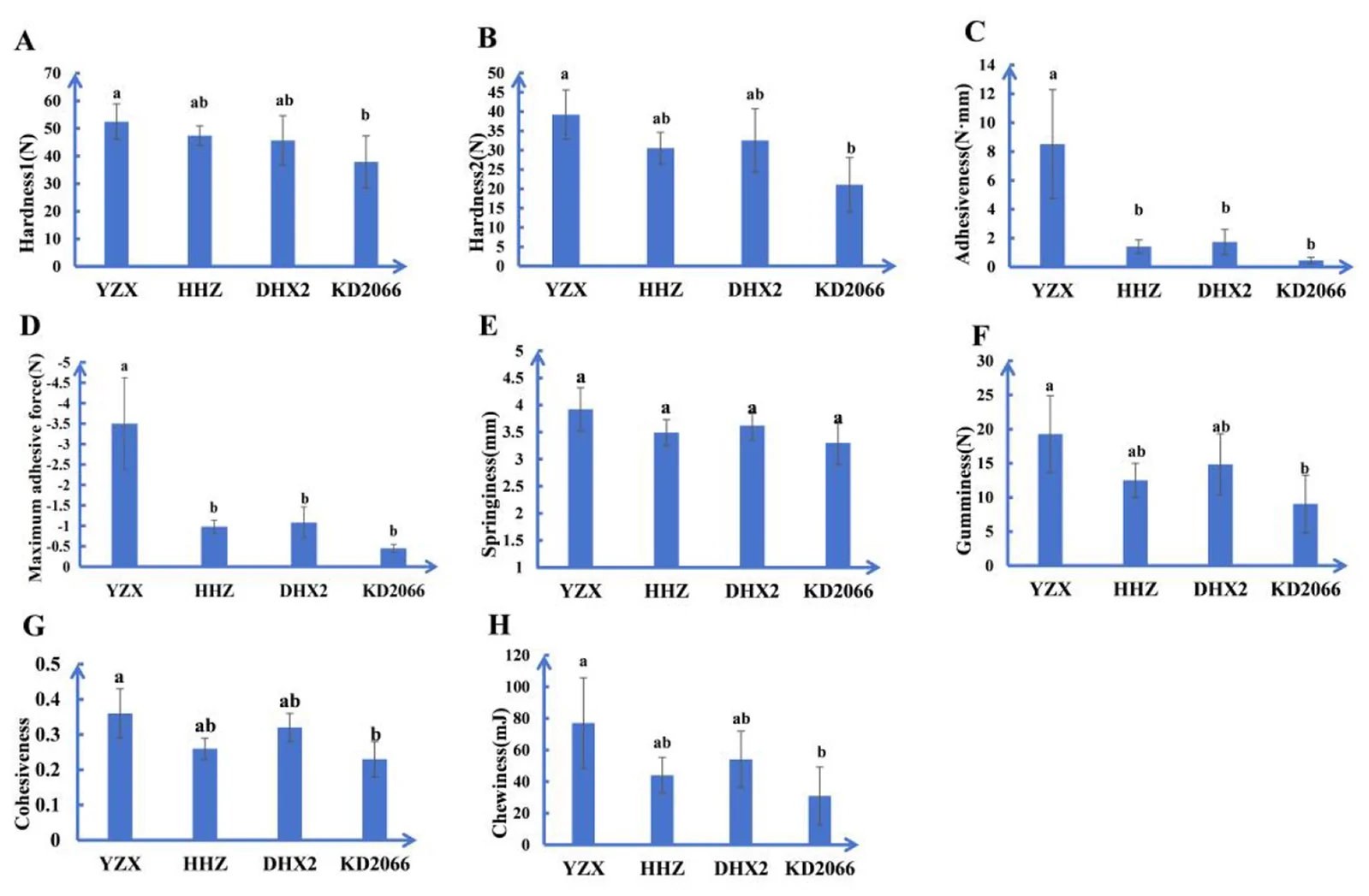
Textural properties of the four rice cultivars. **(A)** Hardness 1, **(B)** Hardness 2, **(C)** Adhesiveness, **(D)** Maximum adhesive force, **(E)** Springiness, **(F)** Gumminess, **(G)** Cohesiveness, and **(H)** Chewiness. Means marked with different lowercase letters indicate significant differences at *P* < 0.05.

The TPA of cooked rice (Figure 2) demonstrated that the adhesiveness and maximum adhesive force of YZX were significantly higher than those of the other three cultivars. Its cohesiveness, gumminess, chewiness, Hardness 1, and Hardness 2 were also significantly higher than those of KD2066, with no significant differences in springiness observed among the cultivars.

Adhesiveness serves as a sensitive index to discriminate rice eating quality (16). The high adhesiveness of YZX was consistent with its characteristic of “smooth surface with moderate viscosity and elasticity” and was closely related to amylopectin content and chain length distribution (17). Cohesiveness reflected the deformation resistance of cooked rice structure (18). The significantly higher cohesiveness of YZX indicated a tighter grain structure with better elasticity. Chewiness is a comprehensive index of hardness, cohesiveness, and springiness. The significantly higher chewiness of YZX compared with KD 2066 contributed to its desirable taste of “chewy but not hard”.

In general, cultivars with superior eating quality (YZX and DHX2) exhibited better performance in adhesiveness, cohesiveness, and chewiness; in comparison, KD2066, with inferior eating quality, exhibited lower values for most textural parameters, with HHZ recorded at the intermediate level. The TPA parameters were consistent with sensory evaluation results, confirming that these indices could effectively quantify the eating quality of cooked rice (19, 20). Accordingly, adhesiveness and chewiness can be regarded as core textural markers for rice palatability evaluation.

#### 3.1.4 Starch pasting properties as determined via RVA analysis

Starch pasting properties of the four cultivars were determined via RVA, with the results shown in Table 2.

**Table 2:** RVA profile characteristics of the four rice cultivars.

RVA parameters		YZX	HHZ	DHX2	KD2066
PV	(cP)	3,035.5 ± 37.48a	2,840.5 ± 27.58b	2,861.5 ± 4.95b	1,945 ± 8.49c
TV	(cP)	1,892.5 ± 58.69b	1,721.5 ± 6.36b	2,095.5 ± 55.86a	1,241.5 ± 21.92c
BD	(cP)	1,143 ± 21.21a	1,119 ± 33.94b	766 ± 60.81c	703.5 ± 13.44d
FV	(cP)	2,840.5 ± 50.20b	2,965 ± 22.63b	3,260 ± 26.87a	2,387.5 ± 13.44c
CS	(cP)	948 ± 8.49c	1,243.5 ± 28.99a	1,164.5 ± 28.99b	1,146 ± 8.49b
SB	(cP)	−195 ± 12.73d	124.5 ± 4.95c	398.5 ± 31.82b	442.5 ± 4.95a
GT	(°C)	79.1 ± 0.57c	87.825 ± 0.53b	74.2 ± 0.48d	90.725 ± 0.04a

Values are mean ± standard deviation. Means followed by different lowercase letters within the same row indicate significant differences at *P* < 0.05.

RVA results demonstrated that PV, reflecting starch gelatinization and swelling capacity, was the highest in YZX. DHX2 and HHZ had similar PV values, whereas KD2066 exhibited the lowest PV. BD, an indicator of starch paste shear stability, ranked YZX > HHZ > DHX2 > KD2066. CS, which indicated the retrogradation tendency of cooled cooked rice, was the lowest in YZX and the highest in HHZ. SB reflected the degree of viscosity recovery, hardening, and retrogradation of cooked rice after starch cooling. YZX displayed a negative SB value, significantly lower than other cultivars, enabling persistent softness of cooled rice. DHX2 also had a low SB value; in contrast, KD2066 possessed the highest SB, corresponding to poor texture after cooling. GT was significantly lower in YZX and DHX2 than in HHZ and KD2066, indicating easier cooking for the former two cultivars.

RVA profiles clearly separated high eating quality rice cultivars from conventional high-yield ones. YZX and DHX2 exhibited ideal RVA traits to form soft, sticky and elastic cooked rice, whereas KD2066 had the poorest quality with low BD plus the highest GT and SB. RVA data were highly consistent with instrumental taste and sensory scores. In short, low SB, low GT, high BD and moderate CS act as core RVA markers for rice with high eating quality.

### 3.2 Analysis of main physicochemical traits of rice cultivars with distinct eating quality

#### 3.2.1 Protein and free amino acid

**Figure 3:**
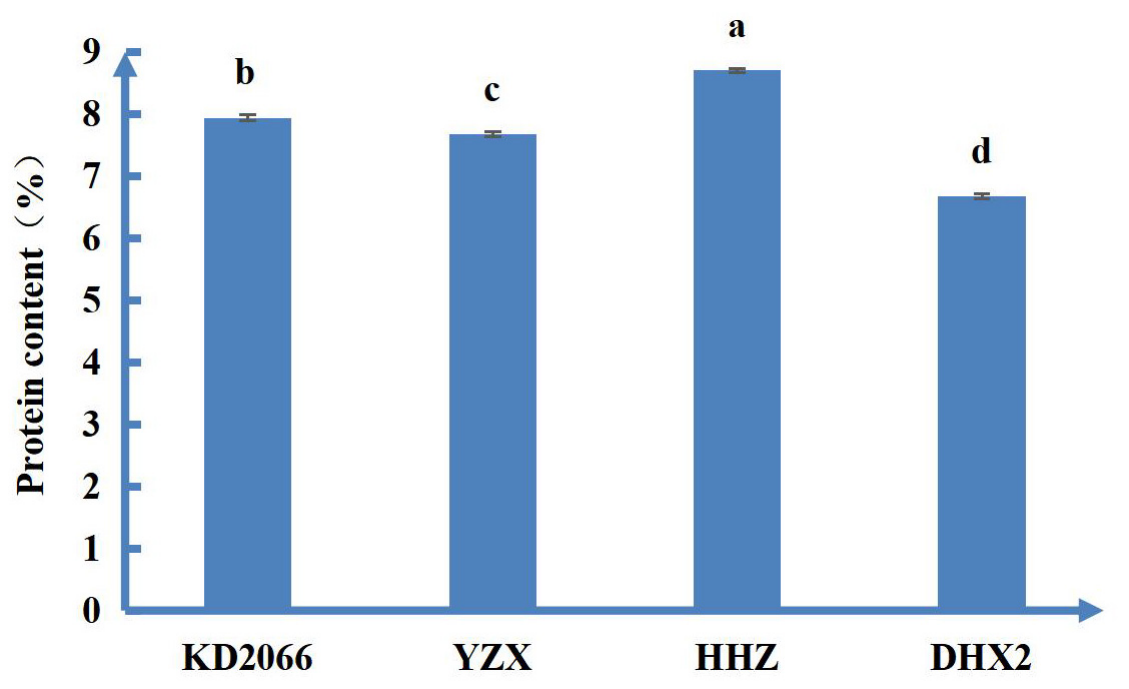
Protein content in milled rice of the four rice cultivars. Means marked with different lowercase letters indicate significant differences at *P* < 0.05.

The protein contents of milled rice from the four cultivars were determined using the Kjeldahl method. Significant differences in protein content were observed among cultivars (*p* < 0.05), with the highest value recorded in HHZ, followed by KD2066 and YZX, and the lowest value (6.68%) detected in DHX2 (Figure 3).

These findings were consistent with those of previous studies, in which a significant negative correlation between rice protein content and eating quality was reported (21, 22). In this study, cultivars with superior eating quality (YZX and DHX2) were found to possess low protein contents, whereas cultivars with higher protein contents (HHZ and KD2066) were associated with moderate eating quality. This phenomenon might be attributed to proteins filling the spaces between starch granules, thereby inhibiting starch gelatinization and expansion and resulting in poorer cooked rice texture.

**Figure 4:**
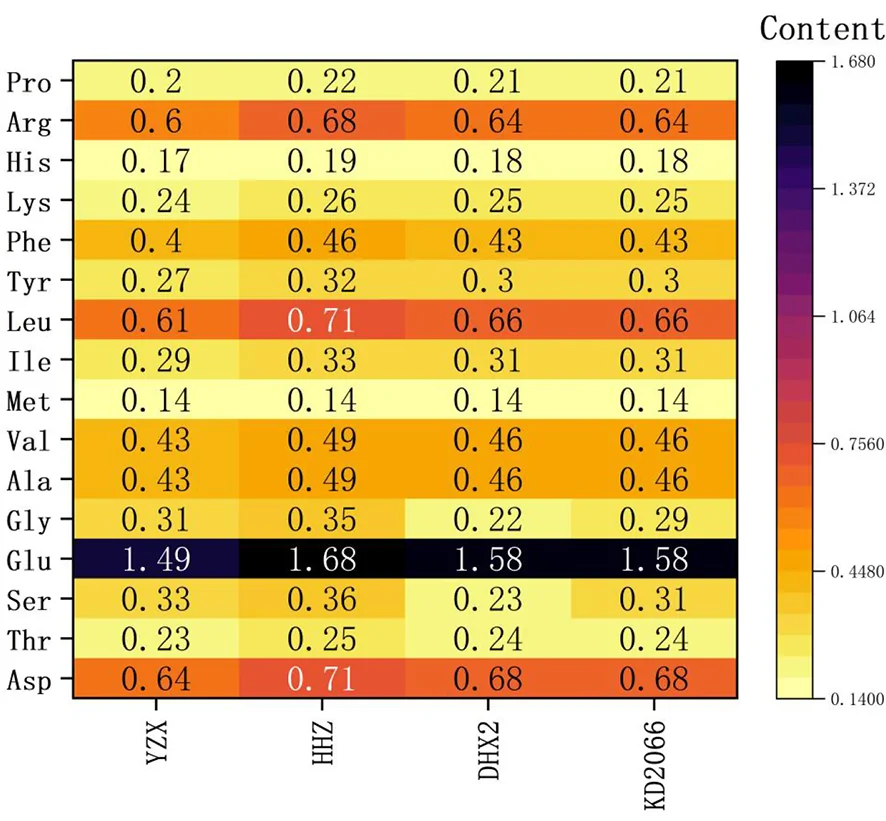
Free amino acid contents (16 types) of four rice cultivars (g/100 g).

Analysis of 16 free amino acids in milled rice of four cultivars demonstrated that glutamic acid (Glu) was the most abundant amino acid (1.49–1.68 g/100 g), whereas methionine (Met) was the least abundant and showed no difference among cultivars (all 0.14 g/100 g). Most amino acids exhibited consistent variation trends across cultivars, with only glycine (Gly) and serine (Ser) exhibiting significantly lower levels in DHX2, resulting in obvious inter-cultivar divergence (Figure 4). Significant differences in total amino acid (TAA) and essential amino acid (EAA) contents were observed among cultivars, both ranked as HHZ > KD2066 > DHX2 > YZX.

The data produced during this experiment, combined with the findings of previous studies, indicated that a low level of free amino acids might represent one of the important biochemical characteristics of rice with superior eating quality. In this study, the high eating quality cultivar YZX exhibited significantly lower contents of TAA and EAA than the other cultivars, which was consistent with the conclusion of Liyanaarachchi et al. (23) and Lu et al. (24), who reported that high eating quality rice cultivars tend to maintain low accumulation levels of free amino acids. Excessive total free amino acid content may be associated with high protein content, which may inhibit starch gelatinization through a physical barrier effect, thereby worsening rice eating quality.

#### 3.2.2 Free sugar content

**Figure 5:**
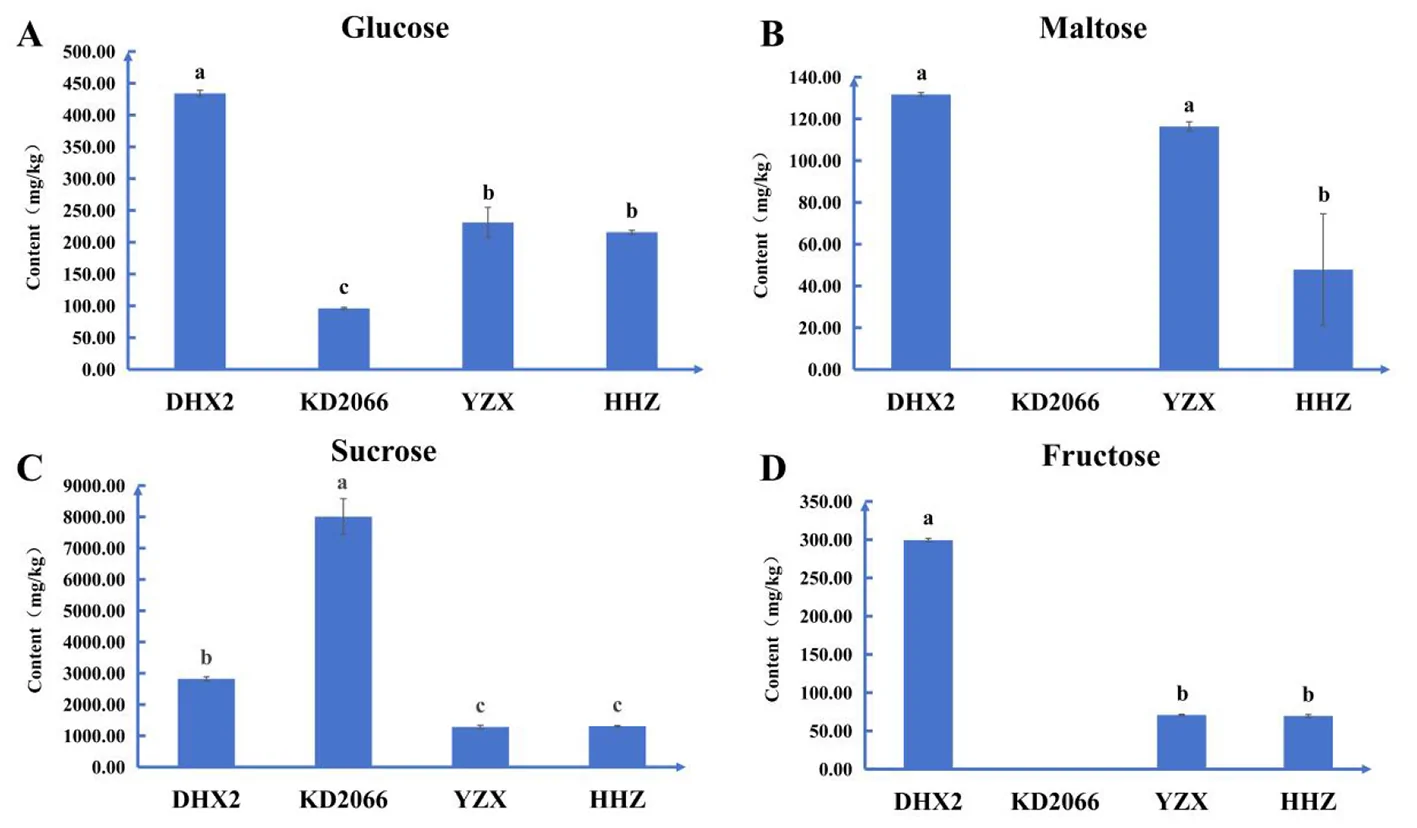
Contents of free sugars in the four milled rice samples (mg/kg). **(A)** Glucose; **(B)** Maltose; **(C)** Sucrose; **(D)** Fructose. Means marked with different lowercase letters indicate significant differences at *P* < 0.05.

Five free soluble sugars (glucose, fructose, maltose, sucrose, and lactose) in milled rice from four rice cultivars were determined via ion chromatography (Figure 5). Lactose was undetectable across all samples, while the composition and concentration of the other four soluble sugars differed markedly among cultivars. DHX2 and YZX exhibited significantly higher maltose contents than the other cultivars, with no statistical difference observed between the two premium cultivars. Moreover, DHX2 exhibited the highest contents of glucose and fructose, which were significantly higher than those of the other three cultivars. KD2066 presented the highest sucrose content but the lowest glucose content, with no detectable fructose and maltose. YZX and HHZ exhibited moderate glucose and fructose, combined with the lowest sucrose content, with no significant differences between the two cultivars.

Free sugar profiles impacted rice eating quality in two aspects: direct taste contribution and aroma formation. Glucose is the main contributor to the sweetness of cooked rice (25), explaining the prominent sweet taste of DHX2 with its elevated glucose concentration. Reducing sugars (glucose and fructose) are key substrates for Maillard and caramelization reactions during cooking, which are critical for aroma development (26). The high reducing sugar level in DHX2 endowed it with greater aroma-forming potential. Maltose was closely related to the aftertaste sweetness of cooked rice, and the high maltose contents in DHX2 and YZX contributed to enhanced sweet aftertaste of cooked rice.

Notably, sucrose displayed a unique variation trend different from other soluble sugars. KD2066, which had the weakest eating quality, possessed the highest sucrose content, while YZX and DHX2 (cultivars with excellent eating quality) as well as HHZ (moderate palatability) maintained relatively low sucrose concentrations. These results suggested that higher sucrose content did not necessarily improve eating quality, with the effect of free sugars on eating quality being a synergistic effect of multiple components. High levels of glucose, fructose, and maltose, combined with moderate sucrose levels, were more conducive to the formation of superior eating quality.

#### 3.2.3 Starch composition and amylopectin chain length distribution

**Figure 6:**
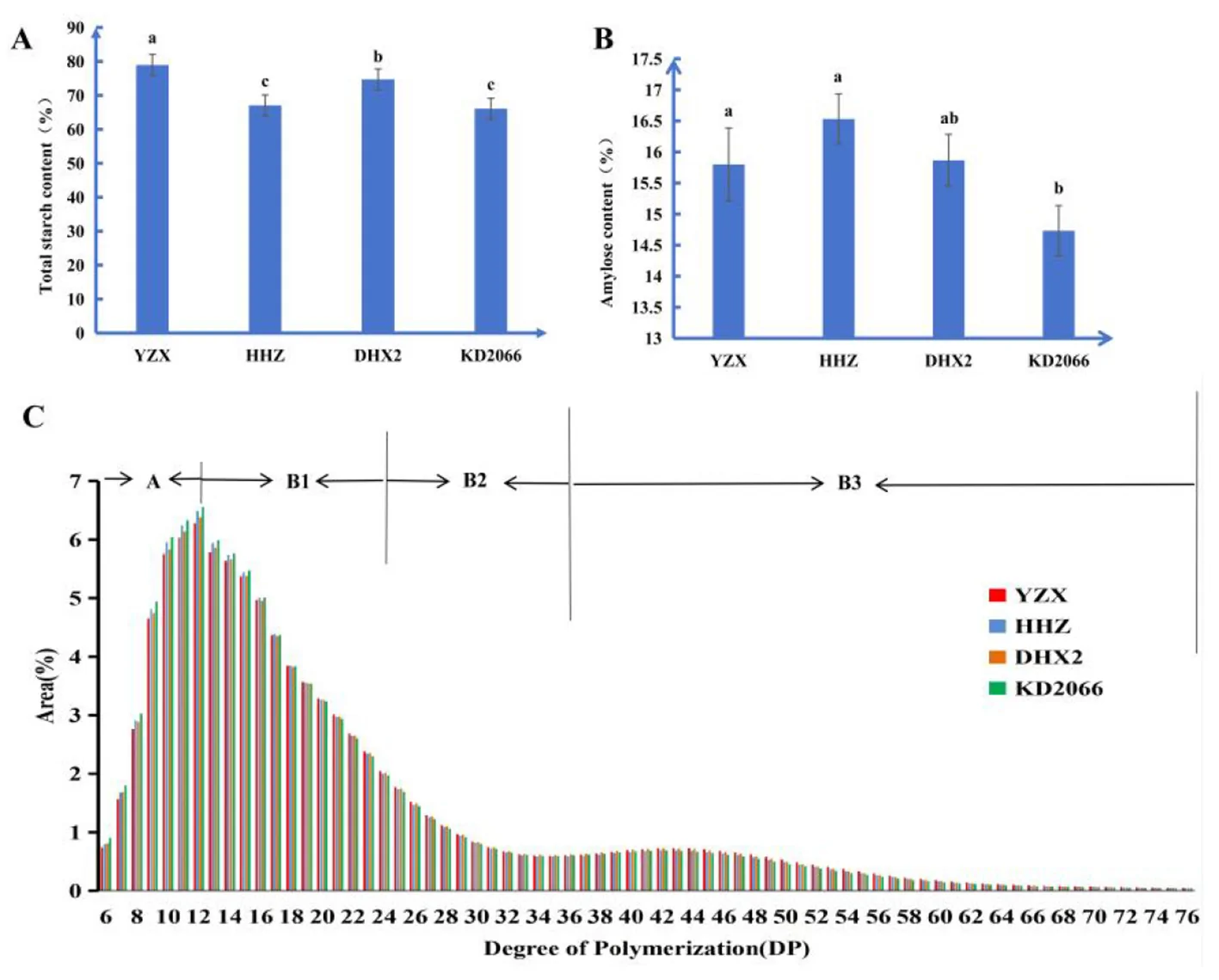
**(A)** Total starch content, **(B)** amylose content, and **(C)** amylopectin chain-length distribution. Means marked with different lowercase letters in panels **(A, B)** indicate significant differences at *P* < 0.05. A, B1, B2 and B3 represent different amylopectin chain populations based on degree of polymerization.

Starch is the main component of rice. Total starch content, amylose content, and amylopectin chain length distribution are coordinately regulated to affect rice eating quality and determine core sensory indicators such as the taste and elasticity of cooked rice. Results of total starch content determined via acid hydrolysis (Figure 6A) showed that YZX possessed the highest total starch content, which was significantly higher than that of the other three cultivars, followed by DHX2. By contrast, HHZ and KD2066 exhibited relatively lower total starch contents. Amylose contents of the four cultivars were determined via spectrophotometry, with the results presented in Figure 6B. Among indica rice cultivars, the amylose content of HHZ was 16.5%, and that of YZX was 15.8%, with no significant difference observed between the two. Among japonica rice cultivars, the amylose content of DHX2 was 15.9%, and that of KD2066 was 14.7%, with no significant difference detected in either case.

These results indicated that amylose content was not the key factor contributing to differences in eating quality within the indica and japonica rice groups in this study. Overall, KD2066 possessed the lowest amylose content among the four cultivars but did not exhibit the optimal eating quality, suggesting that rice eating quality was not regulated solely by amylose content but jointly and coordinately controlled by amylose content and amylopectin chain length distribution. The effect of amylose content on the eating quality of japonica rice was not a simple linear negative correlation; rather, an optimal range was observed. Excessively low amylose content tended to result in overly soft and sticky cooked rice with decreased elasticity, whereas moderate amylose content could endow cooked rice with appropriate hardness and toughness, forming an ideal taste characteristic of soft but non-sticky texture. The results of this study were consistent with the conclusions reported in the previous literature (27).

Amylopectin chain-length distribution acts as a critical structural determinant of rice eating quality. This study further clarified the regulatory mechanism of starch fractions on palatability, providing fine structural evidence to interpret cultivar-specific quality differences that cannot be fully explained by amylose content alone. As indicated by HPAEC-PAD determination results (Figure 6C), the proportions of short-chain fractions (A and B1 chains) of amylopectin in YZX and DHX2 were significantly lower than those in HHZ and KD2066; in comparison, the proportions of medium- and long-chain fractions (B2 and B3 chains) were markedly higher. Specifically, although the amylose content of DHX2 was higher than that of KD2066, its amylopectin was characterized by a lower proportion of short chains, a higher proportion of medium and long chains, and a larger ratio of long to short chains (F(L)/F(S)). The increased rice hardness caused by relatively high amylose content was effectively compensated, and an ideal eating quality was consequently achieved. Consistent with previous studies (28–30), a higher proportion of B2 and B3 medium-long amylopectin chains was strongly correlated with the formation of a more stable spatial network structure of starch molecules, improving the elasticity and cold-storage retrogradation resistance of cooked rice, whereas excessive A and B1 short chains lead to overly sticky rice and severe retrogradation of cold cooked rice. In this study, YZX possessed the highest proportion of medium-long chains (25.90%) and the maximum ratio of long to short chains (0.35), followed by DHX2 (24.71%, 0.33). HHZ (23.97%, 0.32) and KD2066 (23.37%, 0.31) exhibited the lowest values. The chain length distribution characteristics were highly consistent with the eating quality evaluation results of the four cultivars.

In conclusion, rice eating quality depended on the synergistic coordination of total starch content, amylose level, and amylopectin chain-length profile. The superior eating quality of YZX and DHX2 benefited from well-balanced starch structural characteristics, whereas the moderate eating quality of HHZ and KD2066 resulted from unoptimized starch component allocation. Collectively, a low proportion of short amylopectin chains, high proportion of medium and long amylopectin chains, elevated amylopectin long-to-short chain ratio, together with low gelatinization temperature were identified as universal structural indicators for excellent eating quality across both indica and japonica rice, jointly contributing to the desirable soft and elastic texture of high eating quality cooked rice.

#### 3.2.4 Analysis of fatty acid composition

The fatty acid composition and content of milled rice from the four cultivars were determined via GC-MS, and the results are shown in Table 3. All fatty acid components in KD2066 were significantly higher than those in the other three cultivars, resulting in the highest total fatty acid content. HHZ possessed the lowest saturated fatty acid (SFA) content; however, its unsaturated fatty acid (UFA) and linolenic acid contents were higher than those in YZX and DHX2, exhibiting a characteristic of “low saturation and medium-to-high unsaturation”. No significant difference in total fatty acid content was observed between DHX2 and YZX, both of which were significantly lower than KD2066 and classified as low-fatty-acid cultivars.

**Table 3:** Comparison of fatty acid composition contents in four rice cultivars (g/100g).

Fatty acid composition	YZX	HHZ	DHX2	KD2066
SFA	0.0154 ± 0.0007b	0.0114 ± 0.0003d	0.0129 ± 0.0006c	0.0269 ± 0.0006a
UFA	0.0224 ± 0.0012c	0.0288 ± 0.0011b	0.0239 ± 0.0014c	0.0544 ± 0.0023a
Oleic acid	0.0003 ± 0.0001b	0.0005 ± 0.0001b	0.0004 ± 0.0001b	0.0010 ± 0.0001a
Linolenic acid	0.0209 ± 0.0012c	0.0265 ± 0.0008b	0.0219 ± 0.0010c	0.0515 ± 0.0014a

SFA, saturated fatty acids; UFA, unsaturated fatty acids. Values are mean ± standard deviation. Means followed by different lowercase letters within the same row indicate significant differences at *P* < 0.05.

As noted by Guo et al. (31), rice fat content and eating quality exhibit a positive correlation within a certain range, whereas excessively high fat content has a detrimental effect on eating quality. This finding is consistent with the present result that KD2066, with the highest fatty acid content, obtained the lowest eating quality score. Appropriate moderate fatty acid levels help enrich the mild creamy aroma of cooked rice and soften grain texture during chewing. Jia et al. (32) reported that linolenic acid, as a polyunsaturated fatty acid, exhibits poor oxidative stability, and its excessive content tends to accelerate the aging and deterioration of rice. The linolenic acid contents of superior eating-quality cultivars DHX2 and YZX were significantly lower than those of KD2066, which is more conducive to maintaining storage stability and aroma quality. Balanced fatty acid distribution also prevents the formation of stale and rancid off-flavors during long-term rice storage.

#### 3.2.5 Organic acid content

**Figure 7:**
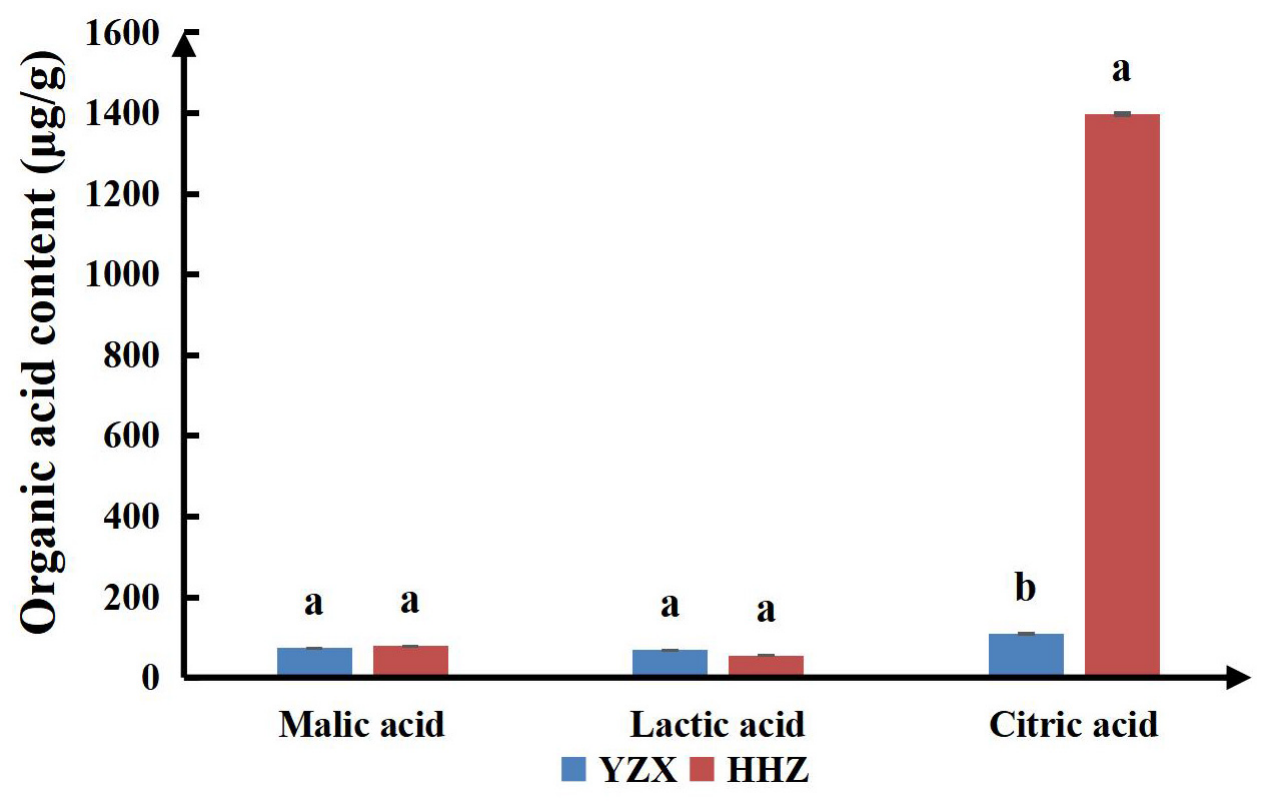
Organic acid contents of YZX and HHZ rice (μg/g). Means marked with different lowercase letters indicate significant differences at *P* < 0.05.

Organic acids are important compounds influencing the refreshing taste and flavor of cooked rice. Appropriate levels can improve eating quality, whereas excessive accumulation often results in sourness-related quality deterioration (33). In the present study, the contents of malic acid, lactic acid, and citric acid in milled rice of the high eating quality cultivar YZX and the conventional high-yield cultivar HHZ were determined, and the results are shown in Figure 7. No significant differences in the contents of malic acid and lactic acid were observed between the two cultivars; in comparison, the citric acid content in HHZ was approximately 12.7 times that in YZX. As reported by Cho et al. (34), excess citric acid lowers grain pH, weakens hydrogen bonding between amylose and amylopectin chains, and accelerates starch recrystallization during cooling; it also produces lingering sour off-notes that mask the subtle natural sweetness and aroma of high-quality rice. The abnormal accumulation of citric acid in HHZ may be one of the key factors responsible for its significantly inferior eating quality compared with YZX, indicating that citric acid can serve as an important metabolic marker for distinguishing rice eating quality. Based on the inter-subspecies metabolic differences of rice summarized in previous research Qi et al. (5), we inferred that superior japonica rice maintains relatively low endogenous citric acid levels during grain filling, while japonica varieties tend to accumulate abundant citric acid similar to HHZ.

#### 3.2.6 2-AP content and aroma quality

**Figure 8:**
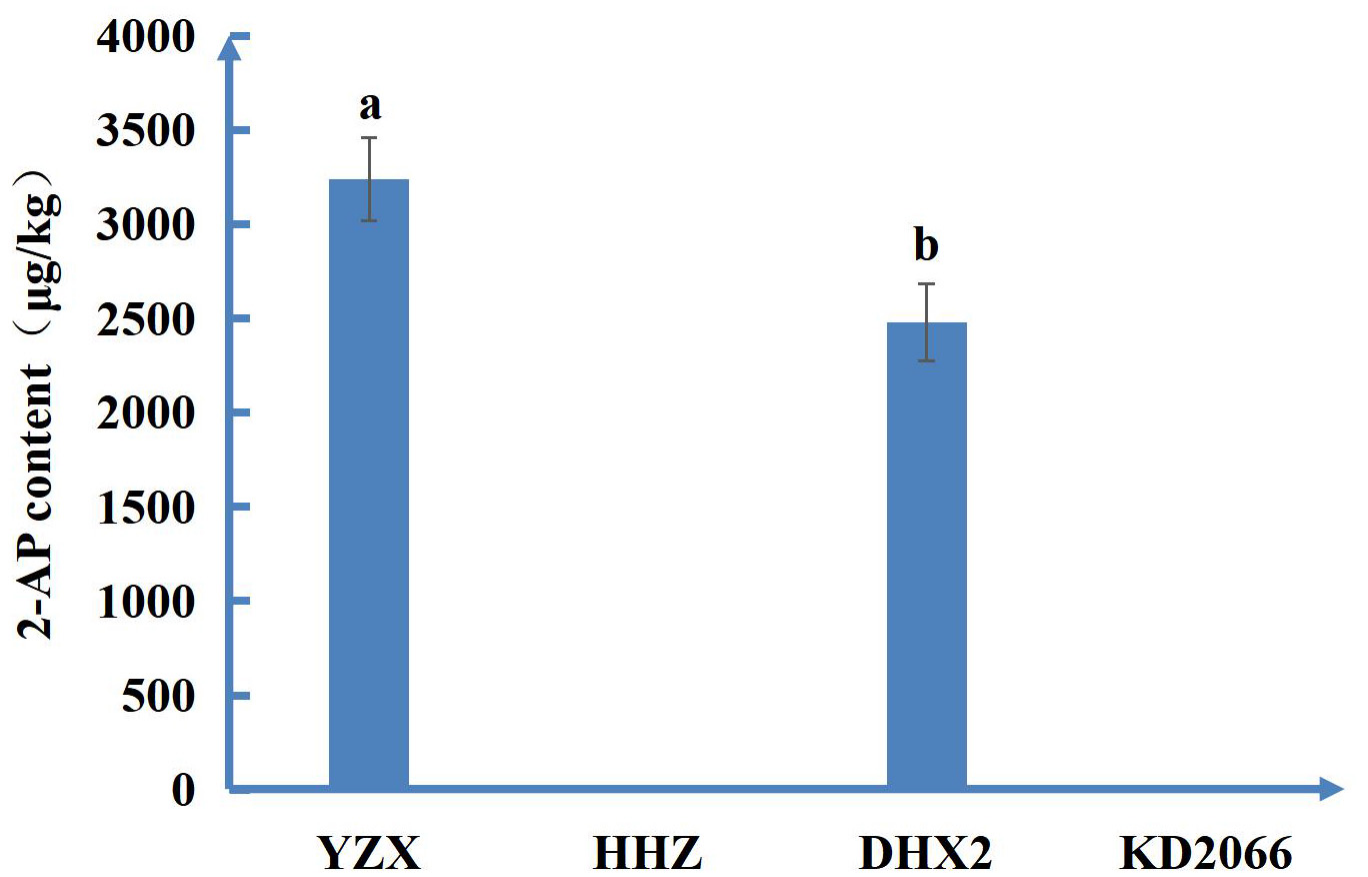
2-AP contents in the four milled rice samples (μg/kg). Means marked with different lowercase letters indicate significant differences at *P* < 0.05.

2-AP is a key volatile compound responsible for the characteristic popcorn-like aroma in fragrant rice, and its content directly determines the aroma quality of rice (35). In this study, headspace solid-phase microextraction coupled with gas chromatography–mass spectrometry (HS-SPME/GC-MS), combined with an internal standard method, was employed to quantitatively analyze the 2-AP content in milled rice of four rice cultivars. The results, shown in Figure 8, indicated that high levels of 2-AP were detected in the fragrant rice cultivars DHX2 and YZX, with contents of 2,479.18 μg/kg and 3,237.24 μg/kg, respectively; by contrast, no 2-AP was detected in the non-fragrant cultivars KD2066 and HHZ.

The results of previous studies by Miao et al. (36) have demonstrated that, although the relative content of 2-AP in the volatile components of cooked rice is only 0.9%−1.6%, it plays a decisive role in the formation of the overall flavor of cooked rice. In the present study, high levels of 2-AP were detected in the superior eating-quality cultivars DHX2 and YZX, findings consistent with their excellent eating quality; by contrast, no 2-AP was detected in the non-fragrant cultivars. These results further verified that 2-AP can serve as a key metabolic marker to distinguish fragrant from non-fragrant rice, in addition to distinguishing high eating quality from conventional high-yield cultivars.

### 3.3 Untargeted metabolomic profiling of YZX and HHZ

#### 3.3.1 Data quality control

**Figure 9:**
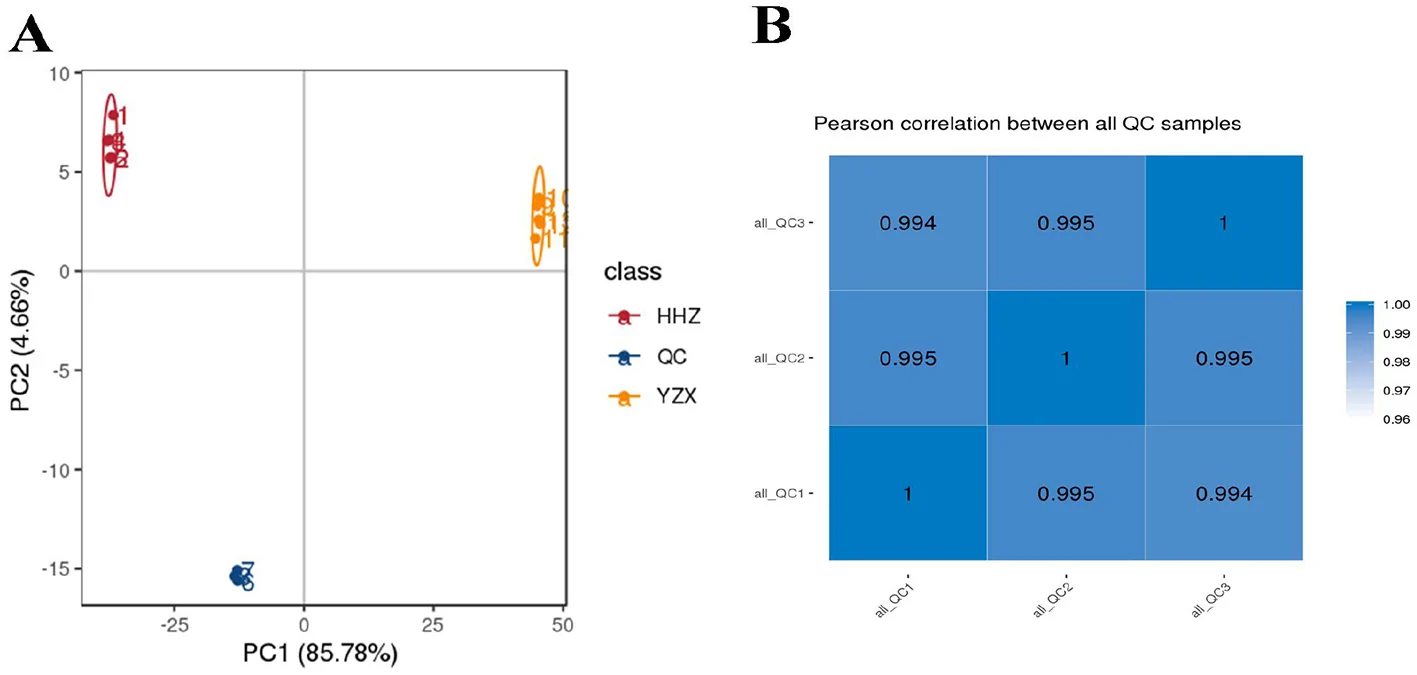
Quality control assessment of metabolomics data: **(A)** PCA score plot of all samples and **(B)** Pearson correlation analysis of QC samples. The abscissa PC1 and ordinate PC2 represent the scores of the first and second principal components, respectively. Scattered dots in different colors denote samples of different experimental groups, and the ellipse indicates the 95% confidence interval.

To evaluate the reliability of untargeted metabolomics data, quality control (QC) samples prepared via equal-volume pooling of all experimental samples were incorporated into the analytical sequence. As shown in the PCA results (Figure 9A), all QC samples clustered closely with a markedly lower dispersion than the experimental samples, indicating good system stability of the instrumental analysis and well-controlled technical errors. Correlation analysis of QC samples revealed that all correlation coefficients were higher than 0.99 (Figure 9B), which demonstrated excellent repeatability of QC samples, stable detection performance, and high quality of the metabolomics data.

#### 3.3.2 Differential metabolite profiling

**Figure 10:**
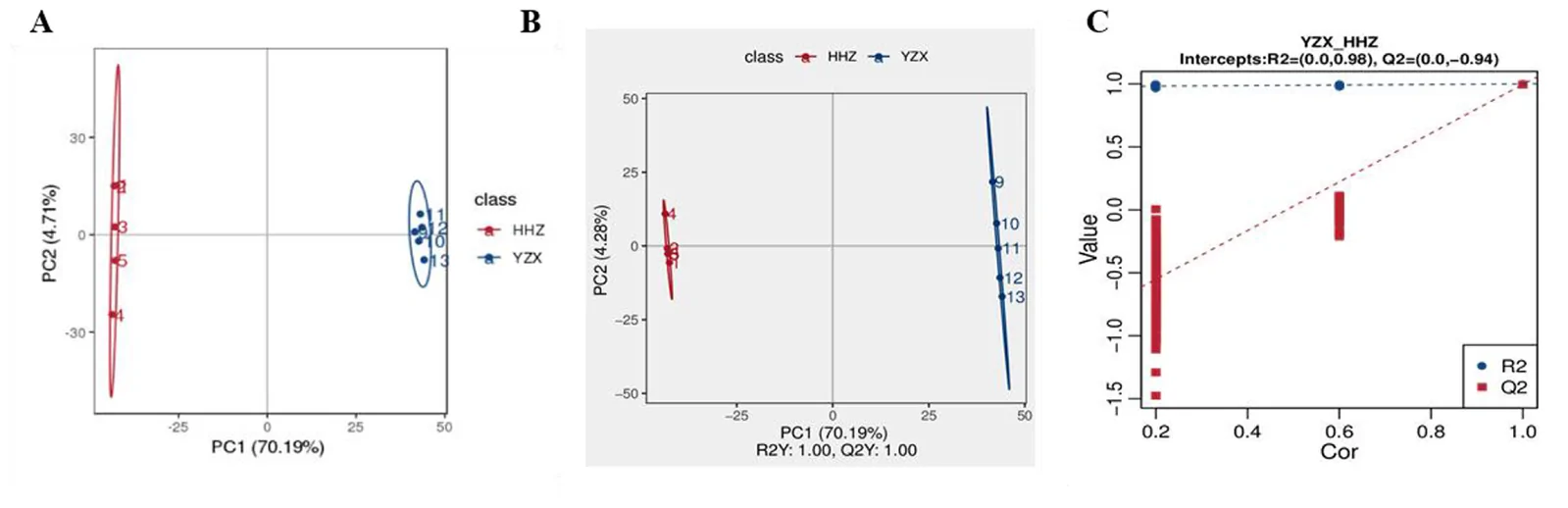
Multivariate statistical analysis of metabolic profiles between YZX and HHZ. **(A)** PCA score plot; **(B)** PLS-DA score plot; **(C)** Permutation validation plot (200 permutations). PC1, principal component 1; PC2, principal component 2.

**Figure 11:**
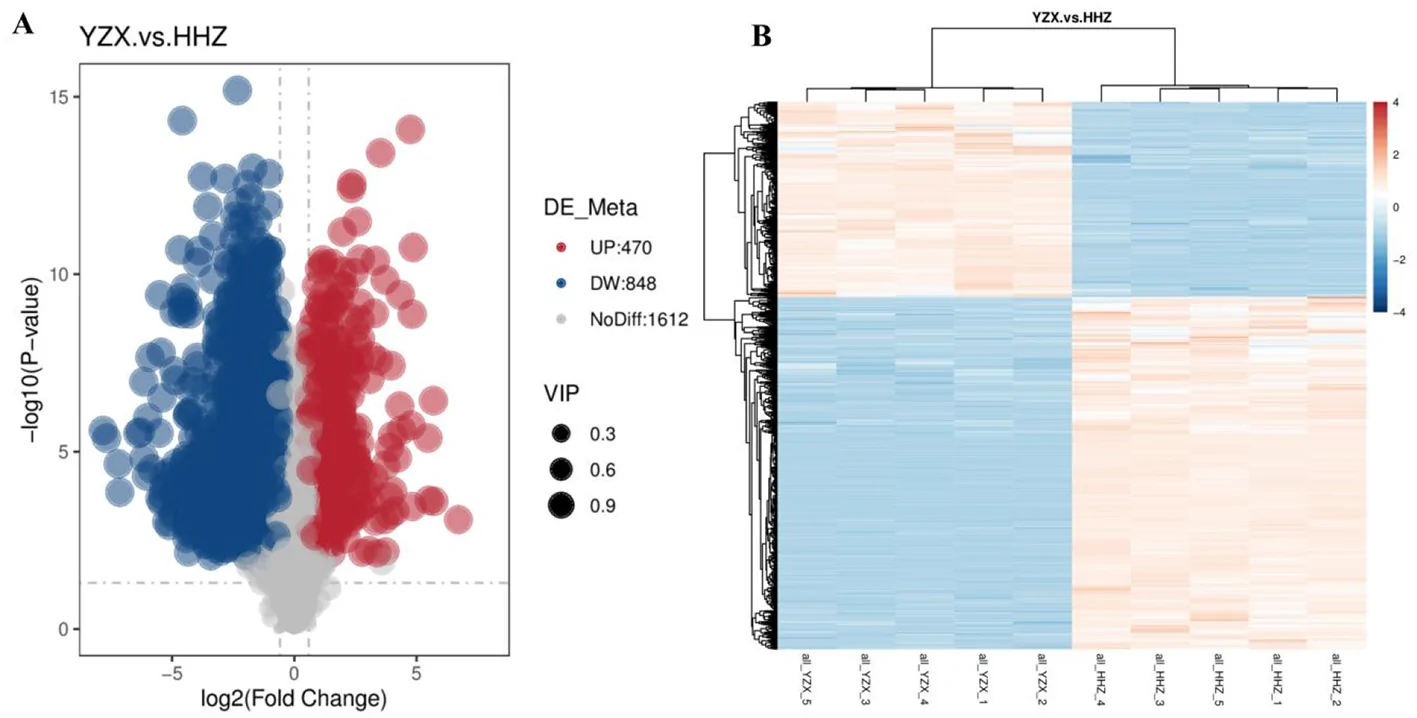
Differential metabolite analysis between YZX and HHZ. **(A)** Volcano plot showing upregulated (red, *n* = 470) and downregulated (blue, *n* = 848) metabolites in YZX. Dashed lines indicate thresholds of VIP > 1.0, |FC| > 1.5 and *P* < 0.05. **(B)** Hierarchical clustering heatmap of 1,318 differential metabolites. Rows represent metabolites, columns represent individual samples. Color intensity corresponds to normalized metabolite abundance (Z-score); red indicates high relative abundance and blue indicates low relative abundance.

PCA and PLS-DA both clearly separated YZX from HHZ, and permutation testing confirmed the robustness of the supervised model (Figure 10), indicating distinct metabolic profiles between the two cultivars. A total of 2,876 metabolites were identified across all samples. Among them, 1,318 metabolites were differentially accumulated between YZX and HHZ (VIP > 1.0, |FC| > 1.5, *P* < 0.05), including 470 upregulated and 848 downregulated metabolites in YZX (Figure 11A), indicating that metabolic differences between the two cultivars were mainly characterized by reduced metabolite accumulation in YZX. Hierarchical clustering of all differential metabolites segregated samples strictly by cultivar with strong intra-group reproducibility (Figure 11B), indicating high reproducibility among biological replicates and consistent metabolic differences between cultivars.

#### 3.3.3 Functional enrichment of differential metabolites

**Figure 12:**
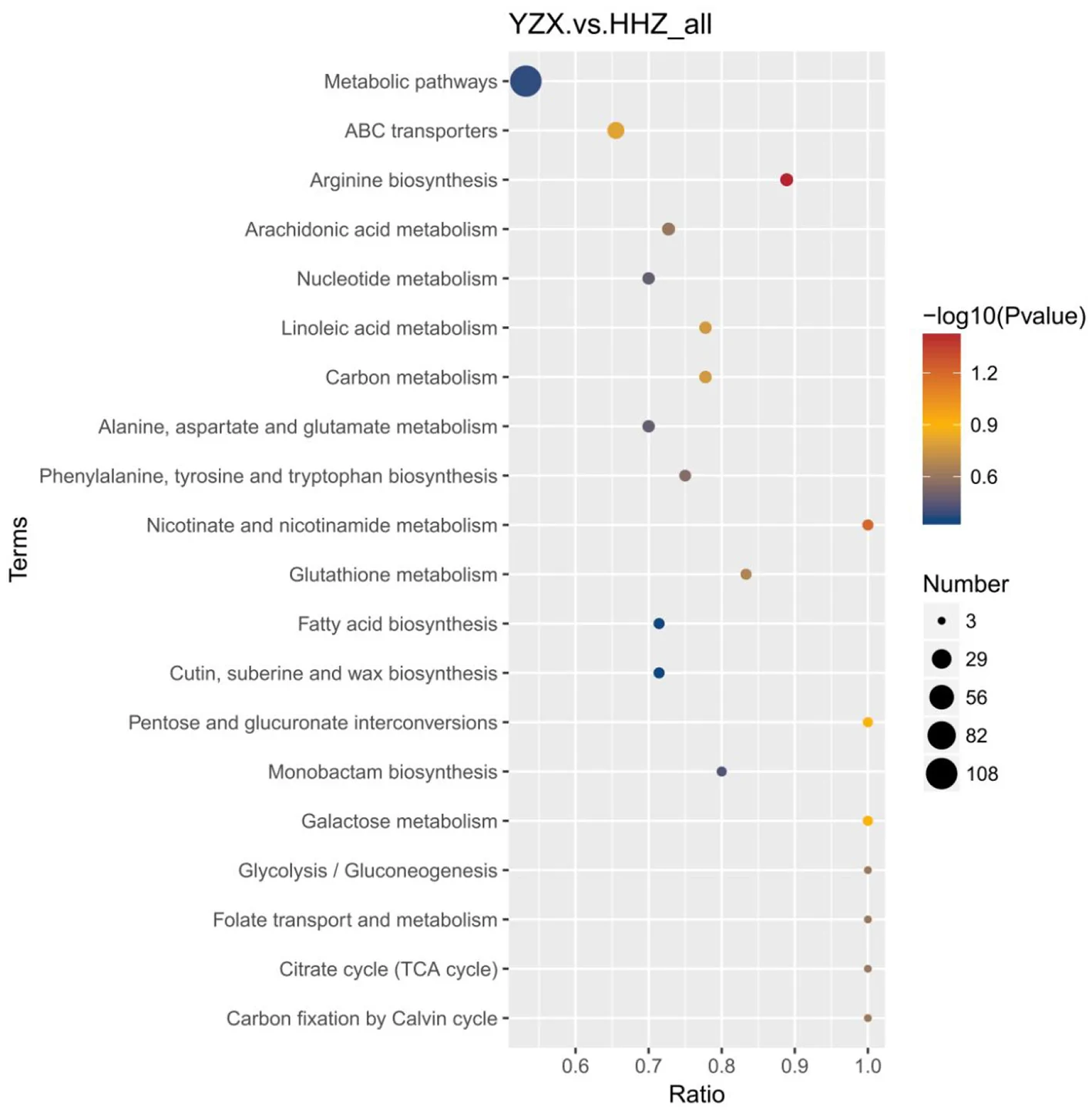
KEGG pathway enrichment analysis of differential metabolites. The x-axis represents the enrichment ratio (Rich Factor). Bubble size corresponds to the number of enriched differential metabolites; color scale indicates the *P*-value calculated from hypergeometric test.

KEGG pathway enrichment analysis mapped differentially abundant metabolites predominantly to amino acid, lipid, and carbohydrate metabolism (Figure 12). Nicotinate and nicotinamide metabolism and arginine biosynthesis were the most significantly enriched pathways (Rich Factor ≈ 1), which might partially explain the metabolic divergence linked to eating quality differences between the two cultivars. Linoleic acid metabolism, arachidonic acid metabolism, phenylalanine/ tyrosine/ tryptophan biosynthesis, carbon metabolism, and galactose metabolism were also enriched, collectively implicating these pathways in flavor compound synthesis, grain texture formation, and basal energy metabolism.

#### 3.3.4 Identification of key metabolites associated with eating quality

Several differentially abundant metabolites with direct relevance to eating quality were identified (Table 4). L-Arginine and L-glutamine were substantially depleted in YZX, indicating reduced nitrogen allocation to amino acid and protein synthesis, consistent with the lower grain protein content of this cultivar. Linoleic acid and its oxidized derivatives (13-HODE, (+)-coronaric acid) were significantly decreased, suggesting attenuated lipid peroxidation and enhanced fatty acid stability conducive to favorable flavor. In contrast, among the key metabolites listed in Table 4, 4-hydroxyproline was the sole prominently upregulated metabolite among the top differential compounds, likely reinforcing cell wall integrity and improving cooking and textural properties. Niacin was significantly reduced in YZX, consistent with the enrichment of nicotinate and nicotinamide metabolism. These coordinated metabolic changes suggested that carbon, nitrogen, and lipid metabolism were closely associated with the superior eating quality phenotype of YZX.

**Table 4:** Key differential metabolites.

Metabolite	Fold change	*P*–value	VIP	Trend
L–Arginine	0.293158828	1.37903E−10	1.193058129	Down
Linoleic acid	0.307664338	1.03287E−13	1.193290619	Down
13–HODE	0.243057678	2.43913E−11	1.192994787	Down
(+)–CORONARIC ACID	0.199265263	6.56864E−16	1.193537334	Down
L–Glutamine	0.272127629	1.30695E−09	1.188722676	Down
4–Hydroxyproline	1.575129899	5.54705E−09	1.190666736	Up
D–Gluconic Acid	0.041810716	4.69460E−15	1.193503337	Down
Trigonelline	0.238425060	1.26955E−08	1.188709232	Down
Niacin acid VB3	0.503538716	1.07963E−10	1.191588770	Down

Fold change: YZX vs. HHZ. Trend “Up” indicates significantly higher abundance in YZX; “Down” indicates significantly lower abundance in YZX.

### 3.4 Correlation analysis and prediction model of rice eating quality

#### 3.4.1 Correlation of multi-dimensional quality indicators

All datasets including sensory evaluation, texture profiles, amylopectin fine structures, grain nutrients and starch pasting properties, as well as correlation data from Supplementary Tables S1–S3, were comprehensively analyzed in this work. Rice eating quality was found to be a complex trait, which was not controlled by a single compound but co-regulated by starch framework, characteristic aroma compounds and small-molecule flavor substances. Common cross-subspecies relationships were validated by correlation analyses. Extremely significant positive correlation (*r* = 0.879^**^) was detected between 2-AP and sensory scores, and typical popcorn aroma of cooked rice was provided by this compound. The proportion of amylopectin B3 chains (*r* = 0.995^**^) and long-to-short amylopectin ratio F (L)/F(S) (*r* = 0.989^*^) both showed extremely significant positive correlations with eating quality scores. These two starch parameters were proven to simultaneously raise adhesiveness (*r* = 0.922^**^) and chewiness (*r* = 0.897^**^) of cooked rice. Extremely significant negative correlation (*r* = −0.842^**^) was observed between RVA CS value and sensory scores, and starch retrogradation was greatly suppressed with low CS values, so soft texture was retained in cooled rice. Eating quality was significantly impaired when protein, free amino acids and CA (*r* = −0.973^**^) accumulated at high levels. Gaps between starch granules were filled by these substances, starch gelatinization was inhibited, and sour off-flavors were generated. Positive correlations were identified between reducing sugars (glucose, fructose) and sensory scores, and balanced sweet taste was produced by these saccharides. Untargeted metabolomic analysis of indica rice identified a series of differential metabolites (Table 4) and supplemented explanatory deficiencies of routine physicochemical measurements. Extremely significant positive correlation (*r* = 0.979^**^) was obtained for 4-hydroxyproline vs. sensory scores; this metabolite improved internal rice structure to achieve elastic and mild sweet taste. Higher contents of linoleic acid (*r* = −0.973^**^) and coronaric acid (*r* = −0.973^**^) were found to accelerate lipid oxidation. Up-regulated L-arginine (*r* = −0.974^**^) was confirmed to promote grain protein synthesis, and rice eating quality was reduced indirectly.

In conclusion, fine starch structures were regarded as the basic substance of rice texture, and 2-AP was determined as the core factor limiting rice aroma quality. Organic acids, amino acids and unsaturated fatty acids were treated as flavor modification regulators. A complete formation pathway of superior palatability suitable for both indica and japonica rice was constructed through the combined effects of the three types of substances. The fragmented research defects of previous reports that only tested single indicators or single rice subspecies were overcome in the present study.

#### 3.4.2 Construction of multiple linear regression model

Based on the above key indicators, a multiple linear regression prediction model was established with sensory eating quality score as the dependent variable (Y) and 2-AP content, the ratio of long to short chain amylopectin (F(L)/F(S)), and consistency viscosity (CS) of RVA as independent variables.


$$\begin{array}{c} \text{Y}=55.663+0.005\times 2-\text{AP}+0.011\times \text{CS}+11.840\times \;\left(\text{F}\left(\text{L}\right)/\text{F}\left(\text{S}\right)\right) \end{array}$$

The model was highly significant (*P* < 0.001) with *R*^2^ = 0.972, meaning the three indicators explained 99.0% of sensory score variation of the present materials. The amylopectin chain ratio had the largest positive coefficient (11.840), contributing most to elasticity and anti-retrogradation. 2-AP positively determined characteristic aroma, while CS reflected cold-rice texture under fixed other factors. Small deviations existed between fitted and measured values of the four cultivars. Residual values between measured and fitted sensory scores were listed in Table 5, ranging from 0 to 1. The small residual range indicated that the regression equation achieved high fitting accuracy for the four tested rice cultivars. The model exhibited weak universality due to limited cultivars, separated single planting areas and narrow germplasm coverage. Future work will expand multi-location germplasm resources and use cross-validation to build a more universal prediction model. It is also worth clarifying our tiered experimental strategy: physicochemical and sensory profiling covering all four cultivars enables cross-subspecies comparison of eating quality phenotypes, whereas metabolomic analysis was restricted to the two indica accessions solely to resolve within-subtype metabolic variation, rather than to compare metabolite profiles between indica and japonica.

**Table 5:** Comparison between fitted values from the regression equation and measured sensory eating quality scores of the four rice cultivars.

Cultivar	2–AP (μg/kg)	CS (cP)	F(L)/F(S)	Fitted value	Measured score	Residual
YZX	3,237.24	948	0.35	86	87	1
HHZ	0	1,243.5	0.32	73	73	0
DHX2	2,479.18	1,164.5	0.33	84	85	1
KD2066	0	1,146	0.31	71	72	1

Residual = Measured score – Fitted value. 2–AP, 2-acetyl-1-pyrroline; CS, consistency viscosity; F(L)/F(S), amylopectin long-to-short chain ratio.

## 4 Conclusion

In this study, four representative indica and japonica rice cultivars with distinct eating quality were subjected to multi-dimensional detection systems including sensory assessment, texture profiling, starch viscosity measurement and multi-component quantification. On this basis, untargeted metabolomics was exclusively performed on two indica cultivars to dissect intrasubtype metabolic divergence. Multi-indicator integrated analysis clarified a unified quality regulatory network applicable to both subspecies: starch structure, 2-AP and small-molecule flavor substances collaboratively govern rice eating quality. High eating quality rice exhibited typical physicochemical traits including low protein, free amino acids and citric acid, moderate soluble sugars, high 2-AP levels, elevated amylopectin long/short chain ratio, low RVA consistency viscosity and gelatinization temperature. A total of 1,318 differential metabolites were identified between the two indica lines, with arginine biosynthesis and linoleic acid metabolism as the dominant divergent pathways. The three-indicator multiple linear regression model (*R*^2^ = 0.972) constructed based on key quality markers can realize preliminary quantitative prediction limited to the tested germplasm. Based on correlation and regression analysis, 2-AP, amylopectin long/short chain ratio and RVA consistency viscosity serve as core shared indicators determining eating quality of both indica and japonica rice. The metabolomic data further supplemented the metabolic mechanisms underlying taste variation only within indica varieties, while the three-factor model only achieves quantitative prediction for the tested germplasm. This study provides multi-dimensional physicochemical and metabolomic evidence, as well as candidate trait markers for high-quality rice breeding and standardized quality evaluation. Future trials with expanded multi-site germplasm are required to validate the universality of these indicators and regression model.

## Data Availability

The original contributions presented in the study are included in the article/Supplementary material, further inquiries can be directed to the corresponding author.
