# Supplementary material for: Physicochemical characteristics and metabolomic profiling of representative rice cultivars distinct in eating quality
Source: Front Nutr. 2026 Aug 17;13:1914365. doi: 10.3389/fnut.2026.1914365 (PMC13527028; doi:10.3389/fnut.2026.1914365)
Supplement: Supplementary file 1 [file Supplementary_file_1.docx]

**TABLE S1** Correlation analysis between various physiological indices and sensory evaluation scores

|  | **sensory evaluation scores** | **Glu** | **AM** | **2AP** | **UFA** | **Glc** | **Fru** | **Suc** | **SFA** | **PKV** | **SB** | **B2 chain** | **F(L)/F(S)** | **B3 chain** | **BD** |
| --- | --- | --- | --- | --- | --- | --- | --- | --- | --- | --- | --- | --- | --- | --- | --- |
| Taste score | 1 |  |  |  |  |  |  |  |  |  |  |  |  |  |  |
| Glu | -0.333 | 1 |  |  |  |  |  |  |  |  |  |  |  |  |  |
| AM | 0.056 | 0.348 | 1 |  |  |  |  |  |  |  |  |  |  |  |  |
| 2AP | 0.879** | -0.657* | 0.022 | 1 |  |  |  |  |  |  |  |  |  |  |  |
| UFA | -0.655* | 0.216 | -0.676* | -0.698* | 1 |  |  |  |  |  |  |  |  |  |  |
| Glc | 0.297 | -0.660* | 0.415 | 0.601* | -0.748** | 1 |  |  |  |  |  |  |  |  |  |
| Fru | 0.144 | -0.719** | 0.305 | 0.489 | -0.595* | 0.977** | 1 |  |  |  |  |  |  |  |  |
| Suc | -0.559 | -0.125 | -0.801** | -0.496 | 0.940** | -0.53 | -0.351 | 1 |  |  |  |  |  |  |  |
| SFA | -0.328 | -0.033 | -0.863** | -0.363 | 0.914** | -0.700* | -0.572 | 0.934** | 1 |  |  |  |  |  |  |
| PV | 0.662* | -0.105 | 0.699* | 0.675* | -0.986** | 0.662* | 0.49 | -0.970** | -0.908** | 1 |  |  |  |  |  |
| CS | -0.842** | 0.286 | 0.336 | -0.741** | 0.219 | 0.082 | 0.196 | 0.149 | -0.154 | -0.265 | 1 |  |  |  |  |
| AP-B2 | 0.844 | -0.635 | 0.35 | 0.961* | -0.874 | 0.744 | 0.64 | -0.691 | -0.617 | 0.835 | -0.623 | 1 |  |  |  |
| F(L)/F(S) | 0.989* | -0.38 | 0.254 | 0.919 | -0.821 | 0.416 | 0.272 | -0.699 | -0.506 | 0.796 | -0.821 | 0.913 | 1 |  |  |
| AP-B3 | 0.995** | -0.358 | 0.219 | 0.908 | -0.794 | 0.368 | 0.223 | -0.676 | -0.47 | 0.77 | -0.845 | 0.892 | 0.999** | 1 |  |
| SB | -0.780** | -0.275 | -0.344 | -0.533 | 0.603* | 0.039 | 0.246 | 0.719** | 0.43 | -0.690* | 0.664* | -0.5 | -0.788 | -0.807 | 1 |

**TABLE S2** Correlation analysis between various physiological indices and sensory evaluation scores

|  | **sensory evaluation scores** | **Glu** | **AM** | **AP** | **2AP** | **UFA** | **Glc** | **Fru** | **Suc** | **SFA** | **PKV** | **SB** | **B2 chain** | **F(L)/F**  **(S)** | **B3 chain** | **BD** | **Hardness1** | **Adhesiveness** | **Chewiness** | **Cor** | **D-GA** | **LA** | **Arg** |
| --- | --- | --- | --- | --- | --- | --- | --- | --- | --- | --- | --- | --- | --- | --- | --- | --- | --- | --- | --- | --- | --- | --- | --- |
| Hardness1 | 0.478 | 0.254 | 0.672* | 0.486 | 0.443 | -.0754** | 0.298 | 0.111 | -0.876** | -0.740** | 0.842** | -0.282 | 0.65 | 0.598 | 0.568 | -0.772** | 1 |  |  |  |  |  |  |
| Adhesiveness | 0.922** | -0.109 | 0.055 | 0.819** | .772** | -0.529 | 0.057 | -0.116 | -0.515 | -0.22 | 0.584* | -0.901** | 0.709 | 0.928 | 0.947 | -0.886** | 0.594* | 1 |  |  |  |  |  |
| Chewiness | 0.897** | -0.273 | 0.388 | 0.894** | .866** | -0.819** | 0.489 | 0.319 | -0.756** | -0.583* | 0.847** | -0.677* | 0.954* | 0.960* | 0.944 | -0.772** | 0.712** | 0.859** | 1 |  |  |  |  |
| Cor | -0.973** | 0.995** | 0.904* | -0.999** | -.999** | 0.960** | -0.929** | -0.256 | -0.362 | -0.976** | -0.980** | 0.996** | -1.000** | -1.000** | -1.000** | 0.999** | -0.333 | -0.985** | -0.942** | 1 |  |  |  |
| D-GA | -0.972** | 0.996** | 0.898* | -0.999** | -.999** | 0.962** | -0.930** | -0.243 | -0.368 | -0.977** | -0.981** | 0.995** | -1.000** | -1.000** | -1.000** | 0.999** | -0.331 | -0.985** | -0.943** | 1.000** | 1 |  |  |
| LA | -0.973** | 0.996** | 0.904* | -0.999** | -.999** | 0.959** | -0.929** | -0.252 | -0.363 | -.977** | -0.979** | 0.996** | -1.000** | -1.000** | -1.000** | 0.999** | -0.331 | -0.985** | -0.942** | 1.000** | 1.000** | 1 |  |
| Arg | -0.974** | 0.997** | 0.901* | -0.999** | -.999** | 0.959** | -0.929** | -0.244 | -0.367 | -.978** | -0.979** | 0.996** | -1.000** | -1.000** | -1.000** | 0.999** | -0.328 | -0.984** | -0.943** | 1.000** | 1.000** | 1.000** | 1 |

**TABLE S3** Correlation analysis between various physiological indices and sensory evaluation scores

|  | sensory evaluation scores | Glu | AM | AP | 2AP | UFA | Glc | Fru | Suc | SFA | PKV | SB | B2 chain | F(L)/F(S) | B3 chain | BD | Hardness1 | Adhesiveness | Chewiness | Cor | D-GA | LA | Arg | Hyp | Hardness2 | Springiness | Breakdown | Mal | CA | Cohesiveness | TS | FAA | PRO |
| --- | --- | --- | --- | --- | --- | --- | --- | --- | --- | --- | --- | --- | --- | --- | --- | --- | --- | --- | --- | --- | --- | --- | --- | --- | --- | --- | --- | --- | --- | --- | --- | --- | --- |
| 4-Hyp | 0.979** | -0.997** | -0.901* | 0.998** | 0.998** | -0.962** | 0.918** | 0.244 | 0.348 | 0.979** | 0.973** | -0.996** | 1.000** | 1.000** | 1.000** | -1.000** | 0.302 | 0.984** | 0.944** | -0.999** | -0.999** | -0.999** | -1.000** | 1 |  |  |  |  |  |  |  |  |  |
| Hardness2 | 0.847** | -0.009 | 0.493 | 0.733** | 0.698* | -0.828** | 0.371 | 0.179 | -0.853** | -0.672* | 0.879** | -0.561 | 0.786 | 0.857 | 0.844 | -0.858** | 0.769** | 0.798** | 0.931** | -0.911* | -0.911* | -0.912* | -0.914* | 0.915* | 1 |  |  |  |  |  |  |  |  |
| Springiness | 0.827** | -0.211 | 0.482 | 0.831** | 0.807** | -0.844** | 0.495 | 0.319 | -0.795** | -0.641* | 0.871** | -0.580* | 0.831 | 0.947 | 0.943 | -0.779** | 0.729** | 0.817** | 0.961** | -0.922** | -0.925** | -0.922** | -0.923** | 0.923** | 0.896** | 1 |  |  |  |  |  |  |  |
| BD | 0.515 | 0.531 | 0.622* | 0.297 | 0.25 | -0.631* | -0.026 | -0.227 | -0.825** | -0.623* | 0.720** | -0.289 | 0.336 | 0.603 | 0.615 | -0.907** | 0.826** | 0.626* | 0.595* | -0.475 | -0.475 | -0.473 | -0.467 | 0.469 | 0.7072** | 0.658* | 1 |  |  |  |  |  |  |
| Mal | 0.719** | -0.672* | 0.266 | 0.892** | 0.915** | -0.847** | 0.860** | 0.765** | -0.635* | -0.627* | 0.796** | -0.422 | 0.984* | 0.827 | 0.799 | -0.374 | 0.481 | 0.541 | 0.810** | -0.992** | -0.990** | -0.992** | -0.991** | 0.990** | 0.670* | 0.779** | 0.223 | 1 |  |  |  |  |  |
| CA | -0.973** | 0.996** | 0.902* | -0.999** | -0.999** | 0.959** | -0.930** | -0.248 | -0.367 | -0.977** | -0.980** | 0.996** | -1.000** | -1.000** | -1.000** | 0.999** | -0.332 | -0.985** | -0.943** | 1.000** | 1.000** | 1.000** | 1.000** | -0.999** | -0.913* | -0.923** | -0.47 | -0.991** | 1 |  |  |  |  |
| Cohesiveness | 0.766** | -0.263 | 0.236 | 0.751** | 0.705* | -0.596* | 0.335 | 0.208 | -0.525 | -0.378 | 0.620* | -0.590* | 0.621 | 0.878 | 0.903 | -0.620* | 0.511 | 0.739** | 0.818** | -0.725 | -0.732 | -0.725 | -0.729 | 0.736 | 0.731** | 0.809** | 0.434 | 0.611* | -0.727 | 1 |  |  |  |
| TS | 0.902** | -0.531 | 0.145 | 0.990** | 0.984** | -0.771** | 0.584* | 0.446 | -0.615* | -0.463 | 0.768** | -0.732** | 0.966* | 0.955* | 0.946 | -0.643* | 0.571 | 0.823** | 0.927** | -1.000** | -1.000** | -1.000** | -1.000** | 0.999** | 0.794** | 0.882** | 0.397 | 0.911** | -1.000** | .760** | 1 |  |  |
| FAA | 0.164 | 0.13 | 0.14 | 0.167 | 0.134 | -0.291 | 0.083 | 0.013 | -0.349 | -0.313 | 0.322 | -0.057 | -0.772 | -0.478 | -0.43 | -0.307 | 0.512 | 0.209 | 0.166 | 0.008 | -0.003 | 0.005 | -0.003 | 0.014 | 0.221 | 0.131 | 0.343 | 0.154 | 0.001 | 0.356 | 0.181 | 1 |  |
| PRO | -0.3 | 0.992** | 0.271 | -0.593* | -0.649* | 0.268 | -0.731** | -0.790** | -0.068 | 0.048 | -0.157 | 0.219 | -0.622 | -0.338 | -0.311 | -0.294 | 0.209 | -0.069 | -0.282 | 1.000** | 1.000** | 1.000** | 1.000** | -0.999** | -0.024 | -0.224 | 0.518 | -0.708* | 1.000** | -0.246 | -0.533 | 0.134 | 1 |

****P<0.01 (two-tailed), extremely significant correlation; *P<0.05 (two-tailed), significant correlation.**

**Note: The English abbreviations of each index in the table are shown as follows.**

Protein content (PRO), Glutamic acid (Glu), Free amino acids (FAA), Total starch (TS), Amylose (AM), Alkali spreading value (ASV), 2-acetyl-1-pyrroline (2AP), Saturated fatty acids (SFA), Unsaturated fatty acids (UFA), Glucose (Glc), Fructose (Fru), Sucrose (Suc), Peak viscosity (PV), RVA consistency viscosity(CS), RVA setback value (SB), RVA breakdown value (BD), Amylopectin B2 chain (AP-B2), Amylopectin B3 chain (AP-B3), Ratio of long to short chains (F(L)/F(S)), (+)-Coronaric acid (Cor), D-Gluconic acid (D-GA), Linoleic acid (LA), L-Arginine (Arg), 4-Hydroxyproline (4-Hyp), Maltose (Mal), Citric acid (CA).
